# Automated Extraction of Pulsatile Stiffness and Wall Asymmetry from Aortic M-Mode Ultrasound Images

**DOI:** 10.3390/bioengineering13070727

**Published:** 2026-06-24

**Authors:** Cheong-Ah Lee, Dong-Guk Paeng, Joon Hyouk Choi

**Affiliations:** 1 Ocean & Biomedical Ultrasound Laboratory, Department of Ocean System Engineering, Jeju National University, Jeju-si 63243, Republic of Korea; cjddk7467@stu.jejunu.ac.kr (C.-A.L.); paeng@jejunu.ac.kr (D.-G.P.); 2 Center of Medicine Platform Based on Smart HemoDynamic Index (SHDI), Seoul 04763, Republic of Korea; 3 Focused Ultrasound Foundation, Charlottesville, VA 22903, USA; 4 Division of Cardiology, Department of Internal Medicine, School of Medicine, Jeju National University, Jeju National University Hospital, Jeju-si 63241, Republic of Korea

**Keywords:** aortic distensibility, aortic stiffness, pulsatile stiffness, automated segmentation analysis, m-mode echocardiogram

## Abstract

Conventional ultrasound-based assessment of aortic stiffness relies on two-point distension metrics using maximum and minimum vessel diameters within a cardiac cycle, which may not fully reflect time-resolved aortic wall dynamics. This retrospective pilot study investigated the feasibility and clinical relevance of a time-series-based stiffness parameter, termed pulsatile stiffness-β, derived from automated segmentation of archived aortic M-mode ultrasound images. Seventy-nine cases with available aortic M-mode images were analyzed. Automated image processing was used to segment the anterior and posterior aortic walls and reconstruct diameter waveforms. Conventional stiffness-β, pulsatile stiffness-β, and wall asymmetry-related parameters were calculated and compared with demographic, tonometry-derived, hemodynamic, coronary burden, cardiovascular risk, and echocardiographic variables. Conventional and pulsatile stiffness-β were strongly correlated and showed directionally consistent associations with established vascular functional parameters, including systolic blood pressure, pulse pressure, augmentation pressure, age, and cardiovascular risk burden. Pulsatile stiffness-β demonstrated association patterns broadly comparable to conventional stiffness-β, suggesting its role as a waveform-informed extension rather than a superior alternative. Wall asymmetry-related parameters were associated with the Syntax score. Automated analysis of archived aortic M-mode images may provide feasible time-resolved vascular biomarkers for stiffness and wall motion assessment.

## 1. Introduction

Aortic stiffness is a well-established marker of vascular aging and an important predictor of cardiovascular morbidity and mortality [1,2,3]. Increased arterial stiffness is closely linked to hypertension, left ventricular overload, impaired coronary perfusion, and adverse cardiovascular outcomes, and it provides prognostic information beyond conventional risk factors [1,4,5]. Although computed tomography and magnetic resonance imaging offer high spatial resolution for vascular assessment, their routine use is limited by cost, accessibility, acquisition complexity, and, in some settings, contrast-related concerns [6,7]. Transthoracic echocardiography therefore remains an attractive noninvasive alternative for evaluating aortic motion in real time. However, conventional echocardiographic assessment of aortic elasticity, particularly M-mode-based assessment, still relies predominantly on maximum and minimum aortic diameters measured at two discrete time points [8,9,10,11]. Accordingly, widely used indices such as aortic strain, distensibility, compliance, and stiffness -β are fundamentally derived from two-point diameter change and may not fully represent the continuous biomechanical behavior of the aortic wall throughout the cardiac cycle.

From a clinical perspective, this limitation is important because arterial function is inherently time dependent [12,13,14]. The arterial waveform over the cardiac cycle reflects not only the magnitude of vessel expansion but also the timing of systolic upstroke, recoil, wave reflection, and ventricular–arterial interaction. Prior hemodynamic research has shown that pulse-wave morphology changes with aging and disease, and that waveform-derived indices contain clinically relevant information related to vascular age and cardiovascular risk [15,16,17,18]. Moreover, increases in pulsatile load and altered wave reflection timing are associated with greater left ventricular afterload, ventricular remodeling, and transmission of excessive pulsatile energy to vulnerable microvascular beds such as the brain and kidneys. These considerations suggest that analyzing only two diameter points may overlook physiologically meaningful information embedded in the temporal pattern of aortic wall motion.

Recent advances in ultrasound acquisition and image-processing techniques have expanded the potential of echocardiography beyond static caliper-based measurements [19,20,21,22,23]. Even though ultrasound has lower spatial resolution than CT or MRI, modern vascular ultrasound methods can track arterial wall motion, evaluate distension waveforms, and enable more localized analysis of wall mechanics over time. These developments create an opportunity to revisit archived M-mode datasets and derive functional vascular biomarkers that were not accessible using conventional two-point measurements alone.

**Figure 1:**
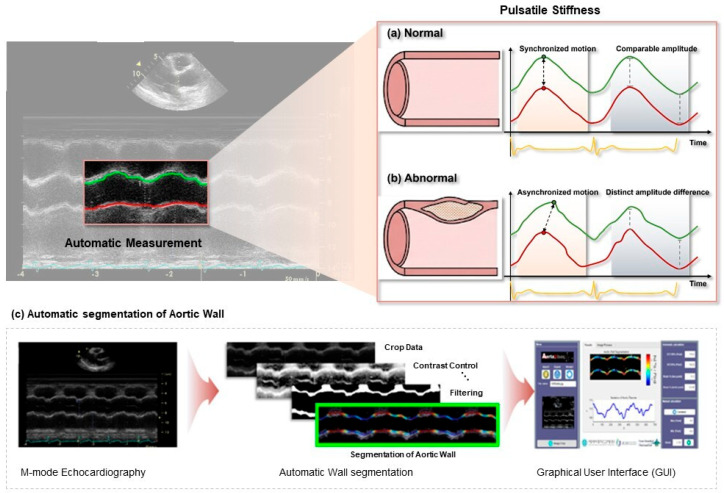
Schematic Chart of the Study Process (Central Illustration). Schematic Chart of the Study Process (Central Illustration). (**a**) A normal aortic wall exhibits syn-chronized motion and comparable amplitude between the anterior and posterior walls, whereas (**b**) an abnormal aorta demonstrates asynchronized motion with a distinct amplitude difference. (**c**) The proposed pipeline processes M-mode echocardiography images through automatic aortic wall segmentation, incorporating crop data, contrast control, and image filtering, with results visual-ized via a graphical user interface (GUI).

Therefore, this retrospective preliminary clinical study was designed to investigate the feasibility and clinical relevance of time-resolved aortic wall dynamics derived from archived aortic M-mode ultrasound images. While conventional stiffness assessment is based on maximum and minimum diameter measurements obtained at two discrete time points, we hypothesized that the pulsatile behavior of the aortic wall over one or more cardiac cycles may provide additional physiological information beyond this simplified framework. To test this concept, we applied an automated image-processing approach to segment the anterior and posterior aortic walls and reconstruct diameter waveforms from retrospective M-mode datasets. Using these waveforms, we defined a time-series-informed stiffness metric, pulsatile stiffness-β, in which the conventional strain-based formulation was extended using a pulsatility ratio to reflect cyclic vascular dynamics. We then evaluated the feasibility of this waveform-informed stiffness parameter by comparing it with conventional stiffness-β and by examining its associations with established hemodynamic, tonometric, and coronary risk indices, as shown in Figure 1. In addition, we explored whether dynamic features derived from time-resolved wall motion, such as wall asymmetry and motion-related behavior, may provide complementary functional vascular information beyond conventional two-point distension-based measures.

## 2. Methods

### 2.1. Patient Population

The study protocol was approved by the Institutional Review Board (IRB) of Jeju National University Hospital, Jeju-do, Republic of Korea (IRB file no. 2022-05-020; approval period: July 2022 to April 2024). Clinical and imaging data were retrospectively obtained from patients who visited Jeju National University Hospital between July 2012 and 11 July 2022. This retrospective preliminary study was not restricted to a specific disease category; instead, cases were selected based on the availability and analyzability of archived aortic M-mode ultrasound images.

A total of 109 archived aortic M-mode image sets were initially screened for analysis. Among them, 30 image sets were excluded, corresponding to an overall rejection rate of 27.5%. Specifically, 19 image sets were excluded because automated segmentation could not be reliably performed due to poorly defined aortic wall boundaries, insufficient ultrasound echo quality, or severe image artifacts. An additional 11 image sets were excluded because the algorithm incorrectly detected valve-related echo signals, resulting in a mismatch between the automatically detected boundary and the true vascular wall location. Consequently, 79 cases were included in the final analytic cohort.

### 2.2. Clinical Variables

Clinical variables for the primary analysis were selected a priori according to their relevance to vascular stiffness, wave reflection, coronary disease burden, and cardiovascular risk. Demographic variables included age, sex, and body mass index (BMI). Hemodynamic and tonometric variables included systolic blood pressure (SBP), diastolic blood pressure (DBP), pulse pressure (PP), augmentation pressure (AP), heart-rate-adjusted augmentation index (Aix@75), and subendocardial viability ratio (SEVR). Coronary atherosclerotic burden was represented by the Syntax score, and global cardiovascular risk was assessed using the estimated Framingham-based total risk score (FRS). Diabetes mellitus (DM) was included as a representative metabolic comorbidity for subgroup comparison. Cardiac functional variables included left ventricular ejection fraction (LVEF) and E/Em. The baseline clinical characteristics of the study cohort are summarized in Table 1. The final analytic population consisted of 79 individuals (48 men and 31 women) with a mean age of 64.6 ± 11.3 years. The mean systolic and diastolic blood pressures were 144.0 ± 19.1 mmHg and 84.9 ± 10.6 mmHg, respectively, and the mean pulse pressure was 59.1 ± 15.8 mmHg. The mean AIX@75 and augmentation pressure were 30.4 ± 9.2% and 18.4 ± 8.6 mmHg, respectively.

### 2.3. Automated Aortic Wall Segmentation

**Figure 2:**
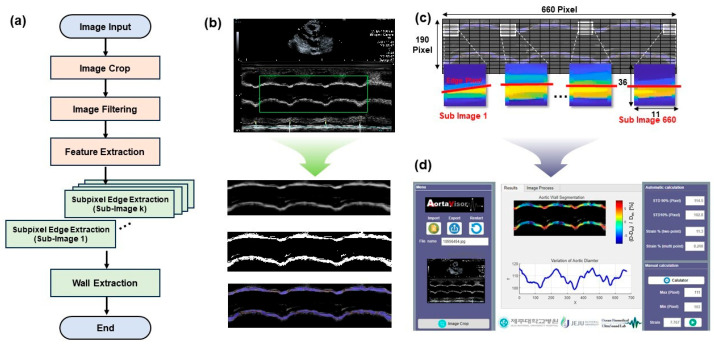
(**a**) A flowchart of the process of extracting information about the dynamic motion of the aortic wall during the cardiac cycle using an automatic image processing algorithm. (**b**) The image processing procedure involves several steps, including selecting a region of interest, applying filtering techniques, removing noise, and binarizing. (**c**) The boundary of the aortic wall is delineated by dividing it into sub-pixels. (**d**) The graphical user interface software displays segmented wall contours, diameter waveforms, and stiffness-related outputs.

The overall image-processing workflow is illustrated in Figure 2a. For image analysis, cropped aortic M-mode images containing the aortic wall region of interest were imported into a custom MATLAB-based graphical user interface (The MathWorks, Inc. (2025). *MATLAB Version 9.18 (R2025b)*. Natick, Massachusetts, United States.). When necessary, the images were converted to grayscale and preprocessed to enhance wall visibility and reduce speckle-related noise, as shown in Figure 2b. Specifically, Gaussian filtering was applied for noise suppression while preserving the global wall-motion pattern, and contrast enhancement was performed to improve boundary delineation. Following preprocessing, the approximate lumen center was estimated from the vertical intensity profile and used as an anatomical reference to separate the search regions of the anterior and posterior aortic walls (Figure 2c). To detect the aortic boundaries, a gradient-based cost map was generated from the filtered image using the vertical image gradient. Intensity-normalized and gradient-weighted terms were combined to define separate cost maps for the upper and lower walls. Spatial constraints were then imposed relative to the estimated lumen center so that the anterior wall was searched only above the lumen center and the posterior wall only below it. The resulting upper and lower wall paths enabled continuous tracking of aortic wall motion over the analyzed M-mode segment and reconstruction of the instantaneous aortic diameter waveform across one or more cardiac cycles. From these spatiotemporal waveforms, dynamic parameters including diameter variation, strain, and stiffness-related indices were calculated. To facilitate practical use, a user-guided software platform was also developed to display the original M-mode image, segmented wall contours, and dynamic waveform outputs (Figure 2d).

To preliminarily assess the reliability of the automated segmentation algorithm, a subset of cases in which both manual and automated measurements were available was subjected to agreement analysis. Two analysts independently measured relative strain from the same archived M-mode images using conventional manual caliper-based approaches, and inter-analyst agreement was evaluated using Bland–Altman analysis and Lin’s concordance correlation coefficient (CCC). Automated relative strain values were subsequently compared with the mean of the two manual measurements using the same framework.

### 2.4. Derivation of Dynamic Aortic Parameters

From the segmented anterior and posterior aortic wall trajectories, the instantaneous aortic diameter waveform D(t) was calculated as the distance between the lower and upper wall positions. Based on this waveform, the maximum diameter ($D_{max}$), minimum diameter ($D_{min}$), mean diameter ($D_{mean}$), and diameter change ($\Delta D=\;D_{max}-D_{min}$) were obtained. Relative strain was calculated as the fractional diameter change during the cardiac cycle, consistent with conventional echocardiographic assessment of aortic elasticity [24].

(1) $$Relative\;strain=\frac{D_{max}-D_{min}}{D_{min}}$$

Conventional stiffness-β was calculated using the logarithmic ratio of systolic to diastolic blood pressure normalized by the diameter-based strain term, in accordance with standard ultrasound-derived arterial stiffness methodology [24,25].

(2) $$\beta =\frac{ln(SBP/DBP)}{Relative\;strain\;}$$

This variable was retained as the conventional reference stiffness parameter in the present study.

In order to incorporate cyclic temporal variability of the diameter waveform beyond two-point diameter extremes, the pulsatility ratio was newly defined in the present study as twice the standard deviation of D(t) divided by the mean diameter. Using this waveform-informed term, pulsatile stiffness-β was defined by replacing the conventional strain term with the pulsatility ratio.

(3) $$\mathrm{pulsatility}\;\mathrm{ratio}=\frac{2\times SD(D(t))}{D_{mean}}$$

(4) $$pulsatile\;stiffness-\beta =\frac{ln(SBP/DBP)}{Pulsatility\;ratio}$$

Because these two metrics were introduced to capture the overall pulsatile behavior of the aortic wall across the analyzed cardiac-cycle segment, they were treated as study-defined dynamic indices without prior direct reference equations in the aortic M-mode literature. The pulsatility ratio was derived from the SD of D(t) calculated across the entire available artifact-free waveform segment, which contained between 2 and 4 cardiac cycles depending on the duration of the archived M-mode sweep. No fixed cycle count was imposed; all artifact-free cycles within the analyzable segment were included in the waveform statistics. For conventional stiffness-β, Dmax and Dmin were identified as the global extrema within the same analyzed segment.

Additional exploratory waveform-derived parameters were also computed, including derivative- and synchrony-based descriptors. After mild smoothing of the diameter waveform, its first temporal derivative (dD/dt) was calculated, and the maximum and minimum values were recorded as indices of systolic expansion rate and recoil slope, respectively.

To quantify the relative balance of motion between the anterior and posterior aortic walls, the extracted wall trajectories were centered relative to their mean positions over the analyzed segment. Because upward displacement of the anterior wall and downward displacement of the posterior wall both correspond to outward vessel distension, the two wall motion signals were reoriented to the same outward-positive direction. The centered anterior and posterior wall motion signals were therefore defined as

(5) $$U_{c}\left(t\right)=-(U\left(t\right)-\bar{U})$$

(6) $$L_{c}\left(t\right)=(L\left(t\right)-\bar{L})$$

where $U\left(t\right)$ and $L\left(t\right)$ denote the instantaneous anterior and posterior wall positions, respectively, and $\bar{U}$ and $\bar{L}$ represent their mean values over the analyzed segment. The motion amplitude of each wall was then quantified using the root-mean-square (RMS) amplitude:

(7) $$A_{u}=\sqrt{\frac{1}{N}\sum_{t=1}^{N}{U_{c}\left(t\right)}^{2}}\;$$

(8) $$A_{L}=\sqrt{\frac{1}{N}\sum_{t=1}^{N}{L_{c}\left(t\right)}^{2}}$$

RMS of the centered wall motion signals were calculated for the anterior wall ($A_{u}$) and posterior wall ($A_{L}$). The wall asymmetry index was defined as ($A_{L}$ − $A_{u}$)/($A_{L}$ + $A_{u}$) and the wall amplitude ratio as $A_{L}$/$A_{u}$. Both parameters were used as exploratory descriptors of asymmetric aortic wall motion.

### 2.5. Statistical Analysis

Statistical analyses were performed to examine the associations between image-derived dynamic aortic parameters and established hemodynamic, tonometric, coronary burden, and cardiovascular risk variables. Continuous variables were summarized as mean ± standard deviation, and categorical variables as counts and percentages. Because several waveform-derived parameters showed non-normal distributions and the present study was exploratory in nature, nonparametric methods were used for the primary analyses.

The main image-derived variables of interest were conventional stiffness-β, pulsatile stiffness-β, wall asymmetry index, and wall amplitude ratio. Associations between these variables and continuous clinical parameters, including age, BMI, SBP, DBP, PP, AP, AIX@75, SEVR, Syntax score, FRS, LVEF, and E/Em, were assessed using Spearman’s rank correlation analysis. Correlation coefficients and two-sided *p*-values were reported.

For subgroup analyses, clinically relevant categorical thresholds were applied. Between-group differences were evaluated using the Mann–Whitney U test for binary variables or dichotomized clinical categories, including diabetes mellitus, age group (<65 vs. ≥65 years), pulse pressure group (<60 vs. ≥60 mmHg), AIX@75 group (below vs. above the cohort median), and Syntax score group (≤22 vs. >22). Comparisons across more than two groups, including Framingham risk strata, were performed using the Kruskal–Wallis test.

Given the exploratory nature of this preliminary study, false discovery rate correction was additionally considered in the interpretation of multiple comparisons. A two-sided *p*-value < 0.05 was considered statistically significant. Emphasis was placed on the direction, magnitude, and physiological plausibility of the observed associations, particularly for stiffness- and wall asymmetry-related parameters.

## 3. Results

The final analytic cohort consisted of 79 cases with archived aortic M-mode images suitable for automated wall segmentation and waveform reconstruction. Time-resolved anterior and posterior wall tracking was successfully completed in all included cases, allowing derivation of conventional stiffness-β, pulsatile stiffness-β, wall asymmetry index, wall amplitude ratio, and additional dynamic waveform-based parameters.

To validate the automated segmentation approach, automated relative strain measurements were compared against those obtained by two independent analysts using conventional manual caliper-based measurements in a matched subset of cases. Inter-analyst reliability of manual relative strain measurements was moderate, with a mean bias of −0.013 (95% limits of agreement [LoA]: −0.127 to 0.103), an intraclass correlation coefficient (ICC) of 0.666, and a Lin’s CCC of 0.663. Comparison of automated versus manual (averaged) relative strain demonstrated a systematic positive bias of 0.160 (95% LoA: −0.117 to 0.438), without significant proportional bias (slope = −0.139, *p* = 0.168). The larger automated strain values likely reflect the methodological distinction between continuous lumen-adjacent wall boundary tracking across the full waveform and the frame-selected two-point caliper measurements used in the manual approach.

**Figure 3:**
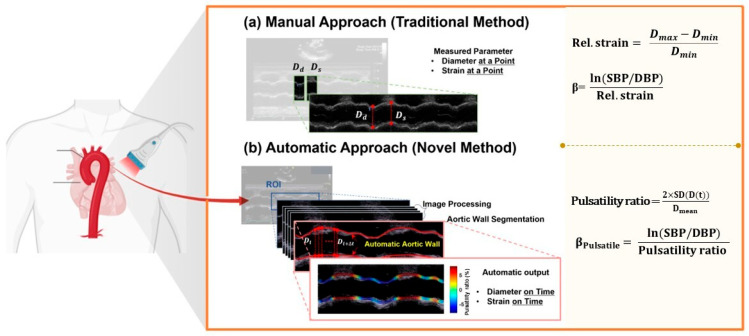
Conceptual comparison between the conventional manual stiffness assessment and the proposed automated time-resolved analysis of aortic wall motion on M-mode ultrasound. (**a**) In the conventional manual approach, the maximum and minimum aortic diameters ($D_{max}$ and $D_{min}$) are measured at a single selected location, from which relative strain and conventional stiffness-β are calculated. (**b**) The proposed automated segments the aortic wall throughout the M−mode image, reconstructs the diameter waveform D(t) over the cardiac cycle, and derives dynamic parameters including pulsatility ratio and pulsatile stiffness-β.

Conventional stiffness-β and pulsatile stiffness-β were strongly correlated (Spearman ρ = 0.940, *p* < 0.001), as illustrated in Figure 3, indicating that both indices reflected a common physiological axis related to arterial stiffness and pulsatile hemodynamics. Pulsatile stiffness-β was consistently higher than conventional stiffness-β in absolute value, which was attributable to the smaller denominator introduced by the revised pulsatility-ratio formulation.

Conventional stiffness-β showed significant associations with age, body mass index, systolic blood pressure, pulse pressure, augmentation pressure, AIX@75, lower SEVR, and Framingham total risk score (Table 2). Pulsatile stiffness-β demonstrated a broadly similar pattern, with significant correlations for age, systolic blood pressure, pulse pressure, augmentation pressure, AIX@75, lower SEVR, Framingham risk percentage, and Framingham total risk points. However, compared with conventional stiffness-β, pulsatile stiffness-β showed numerically weaker correlations with several pressure- and wave reflection-related variables, including systolic blood pressure (ρ = 0.347 vs. 0.398), pulse pressure (ρ = 0.413 vs. 0.448), augmentation pressure (ρ = 0.333 vs. 0.370), and AIX@75 (ρ = 0.254 vs. 0.310).

Wall asymmetry-related parameters showed a distinct association profile from stiffness-related measures. Neither wall asymmetry index nor wall amplitude ratio showed meaningful relationships with age, blood pressure, pulse pressure, augmentation pressure, AIX@75, SEVR, or global risk indices. In contrast, both parameters were positively correlated with the Syntax score (Spearman ρ = 0.334, *p* = 0.003, q = 0.034 for both), as shown in Table 3. This continuous association suggests that wall asymmetry-related parameters may reflect coronary lesion burden in a manner different from pressure-linked stiffness measures.

**Figure 4:**
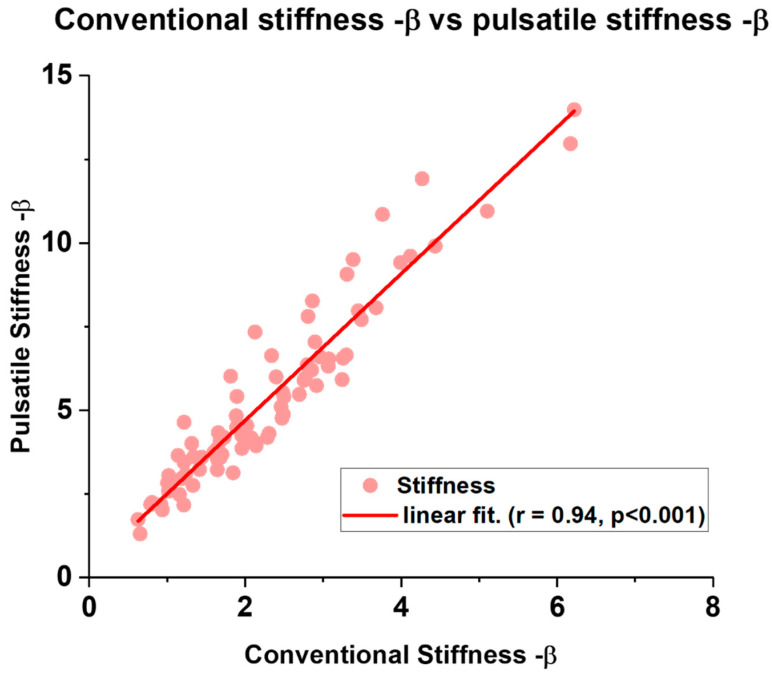
Correlation between the conventional stiffness-β and pulsatile stiffness-β (Spearman ρ = 0.940, *p* < 0.001).

In exploratory subgroup analyses, both conventional stiffness-β and pulsatile stiffness-β were higher in patients with diabetes mellitus, pulse pressure ≥60 mmHg, and higher AIX@75 (Table 4; Figure 4). Pulse pressure showed the clearest separation, with higher median conventional stiffness-β (2.73 [1.82–3.55] vs. 1.70 [1.21–2.40], *p* = 0.001) and pulsatile stiffness-β (5.81 [4.81–7.66] vs. 4.09 [3.07–6.00], *p* = 0.009) in the high-pulse-pressure group. Age-related differences were more modest, with borderline significance for pulsatile stiffness-β (*p* = 0.050). Similarly, subjects with diabetes mellitus and those with higher AIX@75 tended to show higher stiffness values in both formulations, supporting the clinical consistency of the derived waveform-based metrics. When grouped by Framingham risk categories, conventional and pulsatile stiffness-β showed graded increases across risk strata, although these trends did not reach statistical significance (Table 5).

## 4. Discussion

The present retrospective preliminary study has two principal implications. First, it demonstrates that archived aortic M-mode ultrasound data can be repurposed to derive time-resolved vascular biomarkers through automated wall segmentation and waveform reconstruction, thereby extending the clinical utility of conventional echocardiographic archives beyond manual caliper-based measurements. Second, it was specifically designed to explore whether a waveform-informed stiffness parameter, pulsatile stiffness-β, could serve as a clinically meaningful extension of conventional stiffness-β while preserving physiological interpretability. In addition, this study sought to determine whether dynamic features obtainable only from time-resolved wall motion, such as wall asymmetry-related behavior, might provide complementary functional information beyond conventional two-point distension-based measures. In this regard, the present findings support the feasibility of automated time-resolved vascular phenotyping from routine retrospective ultrasound data and provide an initial clinical framework for interpreting these newly derived parameters.

From a methodological perspective, the definition of pulsatile stiffness-β is mathematically plausible because it preserves the core structure of the conventional beta stiffness index while replacing the two-point strain denominator with a normalized waveform-variability term. Conventional stiffness-β is defined as the logarithmic pressure ratio divided by relative diameter strain, and is therefore a dimensionless formulation based on systolic and diastolic diameter extremes. In the present study, pulsatile stiffness-β was constructed by substituting this two-point strain term with a pulsatility ratio derived from the full diameter waveform, thereby retaining dimensional consistency while allowing the denominator to reflect cyclic variability across the cardiac cycle rather than only the maximum and minimum diameters. Although the pulsatility-ratio formulation itself is study-defined and not yet a standardized clinical metric, the rationale for such an extension is supported by the physiological importance of waveform timing and contour in arterial function. Arterial stiffness, wave reflection, ventricular–arterial interaction, and myocardial oxygen supply–demand balance are all influenced not only by the amplitude of vessel distension but also by the temporal distribution of pulsatile behavior [1]. In this sense, pulsatile stiffness-β may be viewed as a mathematically coherent and physiologically motivated extension of conventional stiffness assessment rather than as an arbitrary reformulation.

The present results support this interpretation. Pulsatile stiffness-β remained very strongly correlated with conventional stiffness-β and showed broadly similar relationships with age, systolic blood pressure, pulse pressure, augmentation pressure, AIX@75, lower SEVR, and Framingham-based risk indices. When viewed together, the correlation analysis and subgroup comparisons suggest that the new parameter tracks the same underlying physiological axis of arterial stiffening and pulsatile loading captured by the conventional index. This pattern is meaningful because biological consistency across independent analyses—continuous correlations and clinically interpretable subgroup contrasts—supports construct validity. In other words, the observation that both stiffness indices increased in older subjects, in those with higher pulse pressure, in those with higher AIX@75, and in those with diabetes mellitus is not a trivial duplication; rather, it indicates that the waveform-based reformulation preserves clinically expected risk gradients. At the same time, pulsatile stiffness-β did not consistently outperform conventional stiffness-β, and its correlations with several pressure- and wave-reflection-related variables were numerically weaker. Accordingly, the present data support clinical coherence and feasibility, but not clear superiority. Pulsatile stiffness-β is therefore best interpreted as a waveform-informed extension of conventional stiffness-β that may broaden physiological characterization without displacing the conventional metric.

Another important implication of this work is methodological. Conventional echocardiographic stiffness assessment requires manual identification of maximum and minimum arterial diameters, a process that is time-consuming and potentially vulnerable to observer dependence, especially in retrospective image sets with variable image quality [26]. Automated wall tracking offers an attractive alternative because it allows continuous reconstruction of the diameter waveform and avoids manual selection of only two extrema. More broadly, prior ultrasound studies have shown that automated or semi-automated vessel analysis can reduce operator dependence and improve measurement reliability relative to manual segmentation approaches. Although such evidence comes largely from carotid or brachial applications rather than retrospective aortic M-mode datasets, the general principle is highly relevant here: once the arterial wall can be tracked objectively over time, waveform-based parameters such as pulsatile stiffness-β can be obtained in a more scalable and potentially more reproducible manner than repeated manual caliper measurements. This practical advantage is one of the strongest arguments for continued development of automated time-resolved vascular analysis, even when the new parameters do not yet show clear superiority over conventional indices.

The present findings should nevertheless be interpreted in light of several limitations. This was a single-center retrospective preliminary study with a modest sample size. The pulsatility-ratio formulation was study-defined and, although mathematically coherent, requires external validation and reproducibility testing before broader adoption. Similarly, the segmentation validation presented in this study was conducted at the derived measurement level (relative strain), rather than at the pixel-wise spatial contour level, as frame-by-frame manual contour annotations were not available in the retrospective dataset. The observed systematic positive bias in automated relative strain relative to manual two-point measurements likely partly reflects differences in boundary-selection convention between the two approaches, but the absolute spatial accuracy of the edge detection algorithm cannot be fully characterised from the current dataset alone. Pixel-level validation using in vitro vascular phantoms or prospective frame-by-frame contour annotation will be necessary in future work. Although the pulsatility ratio was adopted as a waveform-derived amplitude index to capture cyclic aortic wall motion, this specific formulation was not selected through comparative optimization. Alternative formulations, such as the coefficient of variation, interquartile range of D(t), or peak-to-peak diameter amplitude, were not evaluated in the present study. Therefore, it remains unclear whether other waveform amplitude metrics would provide better reproducibility, stronger clinical associations, or improved discriminative performance. Future studies should systematically compare candidate waveform-derived indices to identify the most representative and clinically informative denominator for pulsatile stiffness assessment. In addition, stiffness-related indices inherently incorporate blood pressure terms, which may partly explain their close association with hemodynamic variables. The asymmetry-related findings are intriguing, particularly because the wall asymmetry index and wall amplitude ratio were associated with Syntax score, whereas stiffness-related parameters were not; however, this observation remains exploratory, and its mechanistic basis cannot be established in the present design. Additionally, the wall asymmetry index and wall amplitude ratio are susceptible to M-mode probe angulation, as ultrasound reconstructs images from projected acoustic signals, and oblique beam placement can differentially affect the apparent motion amplitudes of the anterior and posterior walls. Although all recordings were acquired in accordance with institutional echocardiographic guidelines using standard parasternal long-axis positioning, subtle inter-acquisition angulation differences cannot be excluded in a retrospective dataset and may represent a confounding factor in wall asymmetry-based indices. Future prospective studies should incorporate standardized probe angulation protocols and formally assess inter-acquisition reproducibility of these parameters. Despite these limitations, the study supports the need for such parameters because conventional two-point distension metrics inherently omit information about waveform shape, temporal variability, and inter-wall motion heterogeneity. Future prospective studies should therefore examine whether waveform-based indices—particularly pulsatile stiffness-β and wall asymmetry-related measures—provide incremental reproducibility, diagnostic value, or prognostic information beyond established stiffness metrics.

## 5. Conclusions

In conclusion, automated analysis of archived aortic M-mode images is feasible for deriving time-resolved vascular biomarkers. Pulsatile stiffness-β showed biologically and clinically consistent relationships broadly comparable to those of conventional stiffness-β, supporting its use as a waveform-informed extension of conventional stiffness assessment. Wall asymmetry-related parameters were additionally associated with coronary lesion burden, suggesting that time-resolved aortic wall dynamics may offer complementary functional information beyond conventional two-point distension metrics. Further prospective studies are needed to validate these findings and define their incremental clinical value.

## Figures and Tables

**Table 1:** Age-based characteristics and demographics.

Characteristic	Total Cohort (*n* = 79)
Demographics	
Age, years	64.6 ± 11.3
Male sex, *n* (%)	48 (60.8)
Body mass index, kg/m^2^	25.2 ± 3.5
Hemodynamic and tonometric variables	
Systolic blood pressure, mmHg	144.0 ± 19.1
Diastolic blood pressure, mmHg	84.9 ± 10.6
Pulse pressure, mmHg	59.1 ± 15.8
Augmentation pressure, mmHg	18.4 ± 8.6
AIX@75, %	30.4 ± 9.2
SEVR, %	154.9 ± 32.8
Coronary disease burden and cardiovascular risk	
Syntax score	8.6 ± 11.2
Framingham risk score, % *	22.8 ± 8.7
Comorbidity	
Diabetes mellitus, *n* (%)	20 (25.3)
Cardiac function	
Left ventricular ejection fraction, %	62.1 ± 12.6
E/Em	12.8 ± 5.8

* Available in 74 patients. Data are presented as mean ± standard deviation or *n* (%).

**Table 2:** Spearman correlations between stiffness-related metrics and selected continuous clinical variables.

Variable	Conventional Stiffness-β	*p*	q	Pulsatile Stiffness-β	*p*	q
Age, years	0.322	0.004	0.008	0.331	0.003	0.008
BMI, kg/m^2^	−0.253	0.024	0.035	−0.227	0.044	0.063
SBP, mmHg	0.398	<0.001	0.002	0.346	0.002	0.008
PP, mmHg	0.447	<0.001	<0.001	0.413	<0.001	0.002
Augmentation pressure, mmHg	0.370	<0.001	0.003	0.333	0.003	0.008
AIX@75, %	0.310	0.005	0.010	0.254	0.024	0.039
SEVR, %	−0.326	0.003	0.008	−0.309	0.006	0.012
Syntax score	0.019	0.865	0.912	0.012	0.918	0.918
Framingham total risk score	0.357	0.002	0.006	0.371	0.001	0.008

Values are Spearman rank correlation coefficients (ρ) with raw *p* values and false discovery rate-adjusted q values. Negative coefficients indicate inverse associations.

**Table 3:** Spearman correlations between wall asymmetry-related metrics and selected continuous clinical variables.

Variable	Wall Asymmetry Index ρ	*p*	q	Wall Amplitude Ratio ρ	*p*	q
Age, years	0.020	0.862	0.991	0.020	0.862	0.991
SBP, mmHg	0.032	0.782	0.991	0.032	0.782	0.991
PP, mmHg	0.001	0.991	0.991	0.001	0.991	0.991
Augmentation pressure, mmHg	−0.031	0.790	0.991	−0.031	0.790	0.991
AIX@75, %	0.019	0.869	0.991	0.019	0.869	0.991
SEVR, %	−0.087	0.448	0.991	−0.087	0.448	0.991
Syntax score	0.334	0.003	0.034	0.334	0.003	0.034
Framingham risk, %	0.002	0.985	0.991	0.002	0.985	0.991
Framingham total risk points	0.023	0.848	0.991	0.023	0.848	0.991

Values are Spearman rank correlation coefficients (ρ) with raw *p* values and false discovery rate–adjusted q values.

**Table 4:** Exploratory subgroup comparisons using Mann–Whitney U tests.

Comparison	Metric	Group 1	Median [IQR]	Group 2	Median [IQR]	*p*	q
DM: no vs. yes	Conventional stiffness-β	*n* = 59	1.89 [1.23–2.83]	*n* = 20	2.49 [2.10–3.25]	0.021	0.042
DM: no vs. yes	Pulsatile stiffness-β	*n* = 59	4.24 [3.17–6.34]	*n* = 20	5.65 [4.45–6.78]	0.045	0.056
Age: <65 vs. ≥65 years	Conventional stiffness-β	<65 (*n* = 37)	1.90 [1.34–2.50]	≥65 (*n* = 42)	2.43 [1.61–3.29]	0.063	0.063
Age: <65 vs. ≥65 years	Pulsatile stiffness-β	<65 (*n* = 37)	4.31 [3.58–5.53]	≥65 (*n* = 42)	5.83 [3.77–7.62]	0.050	0.056
Pulse pressure: <60 vs. ≥60 mmHg	Conventional stiffness-β	<60 (*n* = 41)	1.70 [1.21–2.40]	≥60 (*n* = 38)	2.73 [1.91–3.29]	<0.001	0.008
Pulse pressure: <60 vs. ≥60 mmHg	Pulsatile stiffness-β	<60 (*n* = 41)	4.09 [3.07–6.00]	≥60 (*n* = 38)	5.81 [4.07–7.54]	0.009	0.032
AIX@75: low vs. high	Conventional stiffness-β	<median (30) (*n* = 34)	1.77 [1.19–2.42]	≥median (30) (*n* = 45)	2.48 [1.68–3.24]	0.006	0.031
AIX@75: low vs. high	Pulsatile stiffness-β	<median (30) (*n* = 34)	4.19 [3.07–5.46]	≥median (30) (*n* = 45)	5.74 [3.86–7.34]	0.016	0.040
Syntax score: ≤22 vs. >22	Wall asymmetry index	≤22 (*n* = 67)	−0.06 [−0.15–0.07]	>22 (*n* = 12)	0.11 [0.00–0.17]	0.050	0.056
Syntax score: ≤22 vs. >22	Wall amplitude ratio	≤22 (*n* = 67)	0.89 [0.74–1.16]	>22 (*n* = 12)	1.25 [1.03–1.43]	0.050	0.056

IQR indicates interquartile range. q-values were calculated across the selected subgroup comparisons shown in this table.

**Table 5:** Comparison across Framingham risk categories using the Kruskal–Wallis test.

Metric	<10%	10–<20%	≥20%	*p*	q
Conventional stiffness-β	1.67 [1.34–1.94] (*n* = 10)	2.08 [1.22–2.40] (n = 13)	2.47 [1.64–3.16] (*n* = 51)	0.082	0.165
Pulsatile stiffness-β	3.72 [3.11–4.29] (*n* = 10)	4.31 [3.13–6.20] (n = 13)	5.41 [3.79–7.19] (*n* = 51)	0.070	0.165
Wall asymmetry index	−0.10 [−0.23–0.04] (*n* = 10)	−0.03 [−0.15–0.20] (n = 13)	0.00 [−0.13–0.10] (*n* = 51)	0.486	0.486
Wall amplitude ratio	0.81 [0.63–1.08] (*n* = 10)	0.95 [0.74–1.51] (n = 13)	1.01 [0.77–1.22] (*n* = 51)	0.486	0.486

Values are presented as median [IQR] (*n*). Framingham risk categories were defined using RiskPercent: <10%, 10–<20%, and ≥20%.

## Data Availability

Data shared will be considered on reasonable request to the corresponding author.
